# Impairment of PTX3 expression in osteoblasts: a key element for osteoporosis

**DOI:** 10.1038/cddis.2017.514

**Published:** 2017-10-12

**Authors:** Manuel Scimeca, Antonietta Salustri, Elena Bonanno, Daniela Nardozi, Cecilia Rao, Eleonora Piccirilli, Maurizio Feola, Virginia Tancredi, Annamaria Rinaldi, Giovanni Iolascon, Augusto Orlandi, Elena Gasbarra, Nicola Maffulli, Maria Luisa Brandi, Umberto Tarantino

**Affiliations:** 1 Department of Experimental Medicine and Surgery, University “Tor Vergata”, Via Montpellier 1, Rome 00133, Italy; 2 Multidisciplinary Study of the Effects of Microgravity on Bone Cells” Project, Italian Space Agency (ASI), Spatial Biomedicine Center, Via del Politecnico snc, Rome 00133, Italy; 3 Department of Biomedicine and Prevention, University of Rome “Tor Vergata”, Via Montpellier 1, Rome 00133, Italy; 4 Department of Orthopaedics and Traumatology, “Tor Vergata” University of Rome, “Policlinico Tor Vergata” Foundation, Viale Oxford 1, Rome 00133, Italy; 5 Department of Medicine of Systems, University of Rome Tor Vergata, Via Montpellier 1, Rome, Italy; 6 Department of Medical and Surgical Specialties and Dentistry, Second University of Naples, Naples, Italy; 7 Department of Musculoskeletal Disorders, School of Medicine and Surgery, University of Salerno, Salerno, Italy; 8 Queen Mary University of London, Barts and the London School of Medicine and Dentistry, Centre for Sports and Exercise Medicine, Mile End Hospital, 275 Bancroft Road, London E1 4DG, England; 9 Department of Surgery and Translational Medicine, University of Florence, Florence 50139, Italy

## Abstract

Pentraxin 3 (PTX3) is a multifunctional glycoprotein regulating inflammatory response, cell proliferation and migration and deposition and remodelling of the extracellular matrix by a variety of cells. In this study, we investigated the possible role of PTX3 in bone homeostasis. To this end, we compared the expression and function of PTX3 in human osteoblasts of osteoporotic, osteoarthritic patients and young subjects not affected by bone diseases. Immunohistochemical analysis performed on bone head biopsies showed a close association between bone health and the number of osteoblasts expressing PTX3. Noteworthy, the proportion of PTX3-positive osteoblasts resulted to be significantly lower in osteoporotic patients compared with both young patients and osteoarthritic patients of the same age. *Ex vivo* culture of osteoblasts isolated from the three groups of patients confirmed *in vivo* observation. Specifically, we observed rare runt-related transcription factor 2 (RUNX2) immunopositive osteoblasts expressing PTX3 in cell cultures derived from osteoporotic patients and western blotting analysis showed 80% reduction of PTX3 in the corresponding culture extracts compared with young and osteoarthritic patients. The treatment of human osteoblast primary cultures derived from young patients with anti-PTX3 antibody dramatically affected osteoblast behaviour. Indeed, they lost the morphological and molecular features typical of mature osteoblasts, acquiring fibroblast-like shape and highly decreasing nuclear factor kappa-B ligand (RANKL) and RUNX2 expression. Also, the inhibition of PTX3 negatively affected osteoblast proliferation and their ability to form cell clusters and microhydroxyapatite crystals. Altogether, these results suggest a central role of PTX3 in bone homeostasis showing its involvement in osteoblast proliferation, differentiation and function.

After reaching peak bone mass in the third or fourth decade in life, the bone density begins to decline.^1, 2^ This process accelerates with advancing age resulting in a progressive loss of bone solidness.^1^ Osteoporosis, the most common metabolic bone disease of the elderly, is characterized by a decreased bone strength that significantly increases the risk of fractures.^3, 4^ Indeed, osteoporosis-related fractures are one of the major problems in the elderly population, leading to a significant increase in patient morbidity and consequently in health-care service costs.^5, 6^ From cellular point of view, bone of osteoporotic (OP) patients exhibit an imbalance between the osteoblast and osteoclast activity with the consequent constant decline of bone quality in term of bone matrix composition, structural integrity of each hierarchical length scale (i.e., osteon size and distribution) and microdamage accumulation.^7, 8^ Advances in knowledge of local and systemic factors regulating matrix remodelling as well as the identification of new markers of osteoblastogenesis and osteoclastogenesis are needed for the design of more effective therapies.

Pentraxin 3 (PTX3) is the prototypic long pentraxin first identified in the early 1990s.^9^ Conversely to the short pentraxin C-reactive protein (CRP) and serum amyloid P component (SAP), which are produced primarily in the liver in response to IL-6,^10^ PTX3 is released by peripheral blood leukocytes and myeloid dendritic cells in response to primary pro-inflammatory stimuli by acting as a non-redundant component of the humoral arm of innate immunity and as an essential player in tuning inflammation.^11^ PTX3 is also produced by several stimuli in different cell types, such as vascular endothelial cells, smooth muscle cells, fibroblasts, adipocytes, chondrocytes, mesangial and epithelial and mesenchymal stromal cells.^12^ The main structural determinant of the long pentraxins is the presence of an amino-terminal domain, which is missing in CRP or SAP, coupled to the C-terminal pentraxin domain.^13^ In agreement with the local production and a domain with unique sequence, in addition to its involvement in immunoregulation, PTX3 has been implicated in various other biological processes in physiological and pathological conditions. PTX3 has been found to bind and sequester fibroblast growth factor 2 (FGF2) via its N-terminal extension and to suppress *in vitro* FGF-dependent proliferation of endothelial and smooth muscle cells and *in vivo* tissue neovascularization.^14, 15^ In addition, several lines of evidence have also established a prominent role of PTX3 in extracellular matrix composition and organization. It was recently demonstrated that PTX3 regulates the injury-induced thrombotic response^16^ and promotes wound healing by favouring timely fibrinolysis.^12^ PTX3 expression is also induced by hormones and local factors in the ovary where it has an essential role for assembling hyaluronan^17^ in a matrix suitable for oocyte fertilization.^18, 19^ Few and conflicting data are available to date concerning a possible role of PTX3 in bone metabolism. It has been reported that PTX3 induces the expression of receptor activator of nuclear factor kappa-B ligand (RANKL) by human osteoblasts thereby promoting osteoclastogenesis in an *in vitro* culture system.^20^ On the other hand, preliminary data by Kelava *et al.*^21^ investigated the relationship between PTX3 expression and bone histomorphometry parameters in mice deficient for the PTX3 gene demonstrating that PTX3 null mice had lower bone mass than their WT littermates, implying PTX3 involvement in bone formation.

Based on these evidence, we investigated the possible role of PTX3 in the alteration of bone remodelling that occurs in OP patients. To this end, the expression and function of PTX3 in human osteoblasts of OP patients were compared with those from patients affected by osteoarthritis (OA), that is, of comparable old age but experiencing bone alterations not necessarily linked to bone density loss, and with those from young subjects not affected by bone diseases (CTRL). Analyses were made in both *in vivo* and *in vitro* systems.

## Results

### Clinical evaluation

The OP group included 25 patients with fragility hip fracture, *T*-score ≤−2.5 S.D. and Kellgren−Lawrence (K–L) score from 0 to 1. The OA group included 25 patients with radiographic evidence of hip OA with a K–L score 3 or 4 and *T*-score ≥−2.5 S.D. (Table 1). CTRL patients were characterized by a *T*-score ≥−1.0 S.D. and K–L score from 0 to 1. In addition, OA and OP patients displayed homogenous values of hematochemical exams of both bone and kidney metabolism (Table 1).

### Histomorphometrical analysis

**Figure 1:**
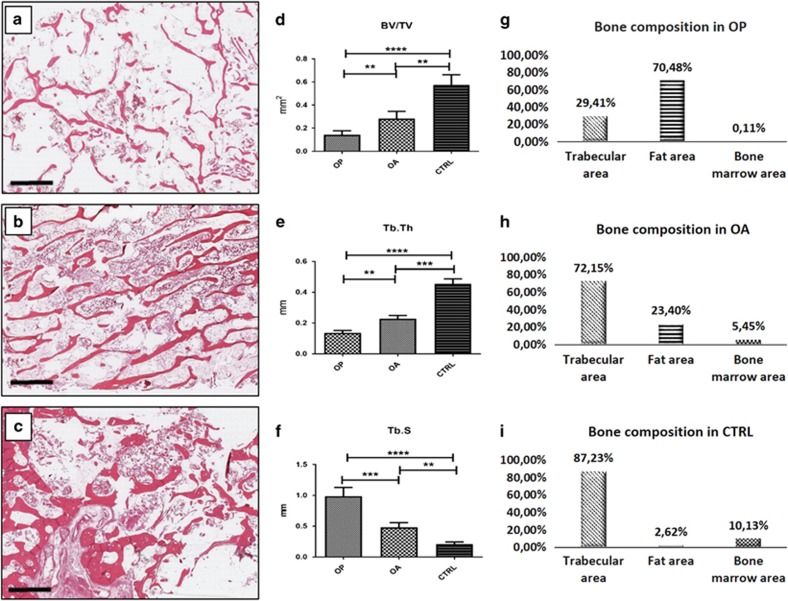
Evaluation of bone morphometric parameters. H&E sections of bone head biopsies (**a**–**c**, scale bar represents 4 mm.). (**a**) OP patients showed a remarkable loss of both trabecular thickness and the number of transversal trabeculae. (**b**) OA patients display moderate trabecular thinning and decrease of transversal trabeculae, (**c**) CTRL patients were characterized by a normal trabecular network. Bone quality parameters evaluated by the BioQuant Osteo software: BV/TV, Tb.Th, and Tb.S (**d**–**f**) One-way ANOVA BV/TV *P*<0.0001, Tb.Th *P*<0,0001, Tb.S *P*<0,0001 (OP *n*=25, OA *n*=25, CTRL *n*=15). Trabecular, fat and bone were calculated by using digital images (**g**–**i**). For each sample, measurements were made at low power magnification

In order to characterize the patients, conventional bone morphometric parameters, such as bone volume (BV/TV), trabecular thickness (Tb.Th) and trabecular separation (Tb.S), were analysed in haematoxylin and eosin (H&E) sections by The BioQuant Osteo software (Figures 1a–f). We detected a significant difference among the patient groups for each considered parameter (*P*=0.0001). For what concerns BV/TV, Mann–Whitney testing showed a significantly lower mean value in OP patients compared with OA and CTRL patients (OP 0.14±0.03 mm^2^; OA 0.28±0.06 mm^2^; CTRL 0.57±0.09 mm^2^; OP *versus* OA *P*=0.0076; OP *versus* CTRL *P*<0.0001, OA *versus* CTRL *P*=0.0031; Figure 1d). Similarly, the mean value of Tb.Th was significantly lower in OP compared with OA and CTRL groups (OP 0.13±0.01 mm; OA 0.23±0.02 mm; CTRL 0.45±0.03 mm; OP *versus* OA *P*=0.0013; OP *versus* CTRL *P*<0.0001; OA *versus* CTRL *P*=0.0001) (Figure 1e). Conversely, Mann–Whitney testing revealed significantly higher Tb.S value in OP patients compared with OA and CTRL patients (Figure 1f; OP 0.98±0.114; OA 0.48±0.08 mm; CTRL 0.21±0.003 mm; OP *versus* OA *P*=0.0040, OP *versus* CTRL *P*<0.0001, OA *versus* CTRL *P*=0.0032).

Bone morphometric analysis allowed us to estimate trabecular, fat and bone marrow areas (Figures 1g–i). OP patients showed a lower percentage of trabecular tissue (29.41%) compared with both the OA and CTRL groups (OA 72.15% CTRL 87.23%) (OP *versus* OA *P*=0.0021; OP *versus* CTRL *P*<0.0001).

In particular, in OP patients the trabeculae were predominantly replaced by adipose tissue (OP 70.48% *versus* CTRL 2.62%), and the bone marrow tissue was significantly reduced (OP 0.11% *versus* 10.13 % CTRL; Figures 1g and i). Conversely, in OA patients, adipose tissue increased much less (23.40 %) and the residual bone marrow area was adequately preserved (5.45 %) (Figure 1h).

### Immunohistochemical analysis of osteoblast markers

We analysed the osteoblast differentiation rate in OP, OA and CTRL patients by immunodetection of specific markers in cells lining the endostium. Immunohistochemical positivity was evaluated on digital images (Iscan Coreo, Ventana, Tucson, AZ, USA) by a semiquantitative approach. We assigned a score from 0 to 3 according to the number of positive osteoblasts on the total analysed for runt-related transcription factor 2 (RUNX2), vitamin D receptor (VDR), osteoprotegerin (OPG) and PTX3 and positive osteocytes for sclerostin. For each sample, we evaluated 50 osteoblasts or osteocytes. The samples containing a number of osteoblasts/osteocytes <50 were excluded by the analysis. Scoring was assigned as reported in Table 2.

**Figure 2:**
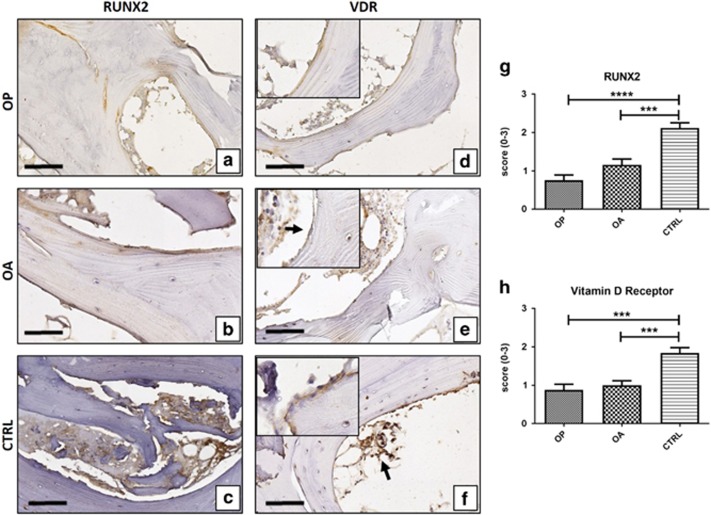
Runx2 and VdR expression. Immunohistochemical results were evaluated by a semiquantitative approach assigning a score from 0 to 3 according to the number of positive osteoblasts (Table 2). (**a**) OP patients showed low expression of Runx2. (**b**) Runx2-positive cells were allocated on trabecular surface (arrow). (**c**) CTRL patients showed numerous Runx2-positive cells both on trabecular surface and bone marrow. (**d**) Cytoplasmic expression of VdR in osteoblasts (square) of OP patients. (**e**) VdR expression in osteoblasts (square 40 × ; arrow) and bone marrow cells. (**f**) CTRL patients showed numerous VdR-positive cells both on trabecular surface (square) and bone marrow (arrow). Scale bar represents 40 *μ*m in all images. (**g**) Graph shows immunohistochemical results of Runx2 (OP *versus* OA *P*=0.4434; OP *versus* CTRL *P*<0.0001; OA *versus* CTRL *P*<0.0001). (**h**) Graph shows immunohistochemical results of VdR (OP *versus* OA *P*=0.4974; OP *versus* CTRL *P*<0.0001; OA *versus* CTRL *P*<0.0001). (OP *n*=25, OA *n*=25, CTRL *n*=15)

In the bone biopsies, we observed that the proportion of RUNX2-positive osteoblasts was not significantly different between OA and OP patients, while in both cases it was lower compared with CTRL (CTRL 2.12±0.13; OA 1.16±0.15; OP 0.76±0.13; OP *versus* OA *P*=0.4434; OP *versus* CTRL *P*<0.0001; OA *versus* CTRL *P*<0.0001; Figure 2).

**Figure 3:**
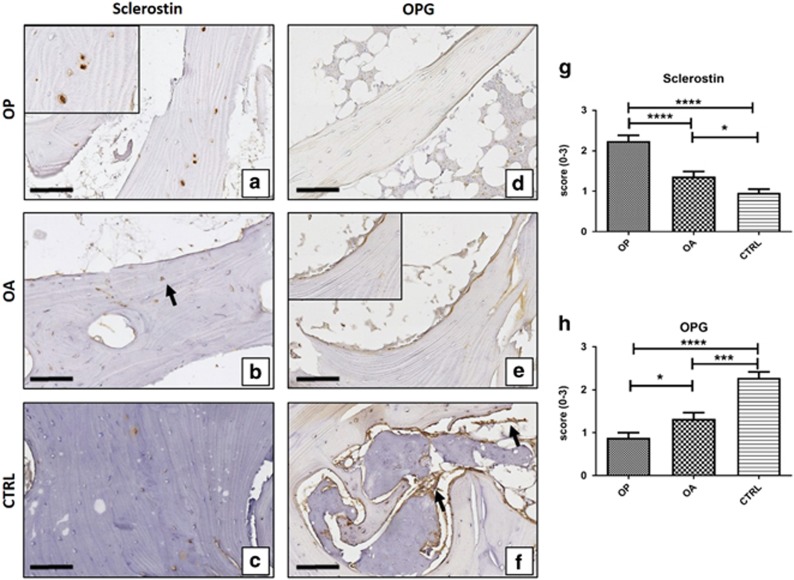
Sclerostin and OPG expression. Immunohistochemistry results were evaluated by a semiquantitative approach assigning a score from 0 to 3 according to the number of positive osteoblasts for OPG and osteocytes for sclerostin (Table 2). (**a**) OP patient showed numerous sclerostin-positive osteocytes (square). (**b**) Trabeculae of a OA patient characterized by both positive (arrow) and negative osteocytes. (**c**) Rare sclerostin-positive osteocyte cells in CTRL patients. (**d**) OP patient with low expression of OPG. (**e**) OPG-positive cells localized on the trabecular surface in a OA patient. (**f**) Numerous OPG positive cells both on the trabecular surface and in bone marrow (arrows) in CTRL patients. Scale bar represents 40 *μ*m in all images. (**g**) Graph shows immunohistochemical results of sclerostin (OP *versus* OA *P*<0.0001; OP *versus* CTRL *P*<0.0001; OA *versus* CTRL *P*=0.0242). (**h**) Graph shows immunohistochemical results of OPG (OP *versus* OA *P*=0.355; OP *versus* CTRL *P*<0.0001; OA *versus* CTRL *P*<0.0001). (OP *n*=25, OA *n*=25, CTRL *n*=15)

Similar results were obtained regarding the expression of VDR (CTRL 1.48±0.14; OP 0.88±0.15; OA 1.00±0.11; OP *versus* OA *P*=0.4974; OP *versus* CTRL *P*<0.0001; OA *versus* CTRL *P*<0.0001; Figure 2). Immunohistochemistry for OPG showed a significant difference among the groups (*P*<0.0001). In particular, we observed a relative lower number of osteoblasts expressing OPG in OP patients compared with OA and CTRL (OP 0.87±0.12; OA 1.32±0.15; CTRL 2.28±0.14; OP *versus* OA *P*=0.0355; OP *versus* CTRL *P*<0.0001; OA *versus* CTRL *P*<0.0001; Figure 3). Also for osteocyte sclerostin expression, we found a significant group difference (*P*<0.0001). As expected, there was a significantly higher relative amount of sclerostin-positive cells in OP patients compared with OA and CTRL patients (OP 2.24±0.14; OA 1.36±0.13; CTRL 0.96±0.09; OP *versus* OA *P*<0.0001; OP *versus* CTRL *P*<0.0001; OA *versus* CTRL *P*=0.0242; Figure 3). These data are in agreement with a decreased osteoblast differentiation in OP patients.

### *In vivo* PTX3 expression and bone metabolism

**Figure 4:**
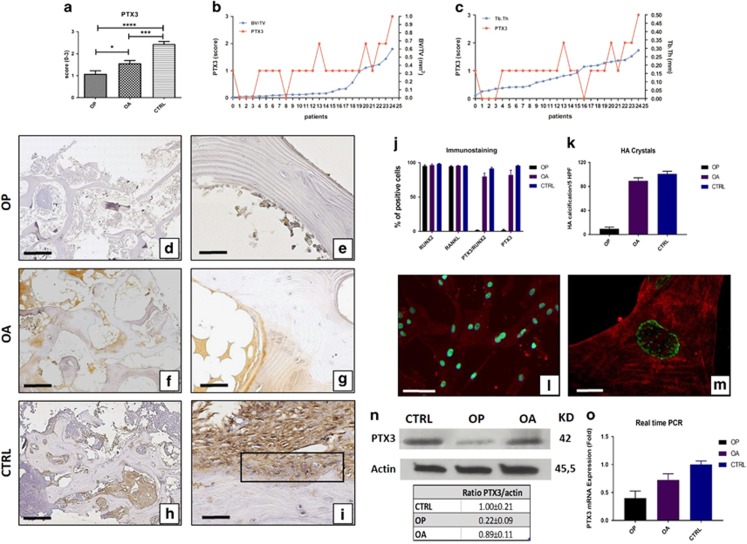
Evaluation of PTX3 in bone head biopsies and osteoblast cell cultures. Immunohistochemical results were evaluated by a semiquantitative approach assigning a score from 0 to 3 according to the number of positive osteoblasts (Table 2). (**a**) Graph shows immunohistochemical results of PTX3 (OP *versus* OA *P*=0.0140; OP *versus* CTRL *P*<0.0001; OA *versus* CTRL *P*<0.0001). (OP *n*=25, OA *n*=25, CTRL *n*=15) (**b** and **c**) Graphs displays the correlation between PTX3 score and BV/TV or Tb.Th values for each OP patient. OP patients are numbered in increasing order of BV/TV or Tb.Th values, respectively. (**f** and **g**) OA patients showed high expression of PTX3 both in osteoblasts and bone marrow cells. (**h** and **i**) CTRL patients showed expression of PTX3 in almost all osteoblasts and bone marrow cells (**g**, square). Scale bar represents 200 *μ*m for DFH images and 40 *μ*m for EGI images. (**j**) Graph shows the results of immunostaining of primary confluent osteoblast cultures (OP *n*=3, OA *n*=3, CTRL *n*=3) (**k**) Graph shows the presence of micro-HA crystals in primary confluent osteoblast cultures (OP *n*=3, OA *n*=3, CTRL *n*=3). (**l** and **m**) Dual-colour immunostaining for PTX3 (red) and RUNX2 (green) on confluent osteoblasts culture derived from CTRL patients. (**l**) Images show numerous RUNX2-positive osteoblast expressing PTX3. Scale bar represents 50 *μ*m. (**m**) High magnification image of RUNX2-positive osteoblast expressing PTX3. Scale bar represents 10 *μ*m. (**n**) Representative images of western blotting assay. Western blotting analysis displayed remarkable difference in PTX3 expression among the groups. Table reported the ratio between the densitometric values of PTX3 and *β*-catenin for each experimental group. (OP *n*=3, OA *n*=3, CTRL *n*=3). (**o**) mRNA expression level of PTX3 relative to GADPH expression in osteoblast primary cultures derived from OP (0.39±0.22), OA (0.72±0.19) and CTRL (1.00±0.11) patients. (OP *n*=3, OA *n*=3, CTRL *n*=3)

Immunohistochemical analysis of biopsies revealed that PTX3 was expressed in human osteoblasts of CTRL. Noteworthy, the relative number of PTX3-positive osteoblasts was significantly lower in OP compared with OA and CTRL patients (OP 1.08±0.14; OA 1.56±0.13; CTRL 2.44±0.11; OP *versus* OA *P*=0.0140; OP *versus* CTRL *P*<0.0001; OA *versus* CTRL *P*<0.0001; Figure 4).

In addition, we plotted PTX3 score and BV/TV or Tb.Th values for each OP patient. We numbered OP patients in increasing order of BV/TV or Tb.Th values, respectively. Interestingly, we found that the few OP patients with relative elevated number of PTX3-expressing osteoblasts, that is, score of 2 or 3, showed higher values of BV/TV and Tb.Th (Figures 4b and c). Altogether, these results suggest a correlation between bone density and PTX3 expression.

### *In vitro* study of PTX3 expression

We then isolated osteoblasts from the cancellous bone of OP, OA and CTRL patients and cultured them *in vitro*. In all cases, after 4 weeks of culture, cells became confluent and >95% were immunopositive for RUNX2 (Figure 4j). Osteoblasts isolated from CTRL and OA patients were characterized by the presence of numerous micro-HA crystals, whereas primary osteoblast cultures derived from OP showed a poor propensity to form micro-HA crystals (Figure 4k). Dual-colour immunofluorescence reaction showed that PTX3 was expressed by >75% of RUNX2-positive osteoblasts in primary osteoblast cultures derived from both OA and CTRL patients (Figures 4j, l and m). Conversely, we observed rare RUNX2-positive osteoblasts expressing PTX3 in cell cultures derived from OP (Figure 4j). This result was confirmed by western blotting analysis with PTX3 antibody. Protein extracts of OP, OA and CTRL osteoblast cultures showed a positive band at about 42 kDa corresponding to the molecular weight of monomeric PTX3. Noteworthy, the signal was remarkably less intense in OP than in OA and CTRL samples, corresponding to almost 80% reduction after normalization for housekeeping protein actin (Figure 4n). At the genomic level, we also demonstrated the decrease of PTX3 mRNA in osteoblast derived from OP patients with respect to both osteoblasts of OA and CTRL by real-time PCR (Figure 4o).

These *in vitro* results further support the evidence of impaired PTX3 expression in osteoblasts of OP patients.

### *In vitro* study of PTX3 function

**Figure 5:**
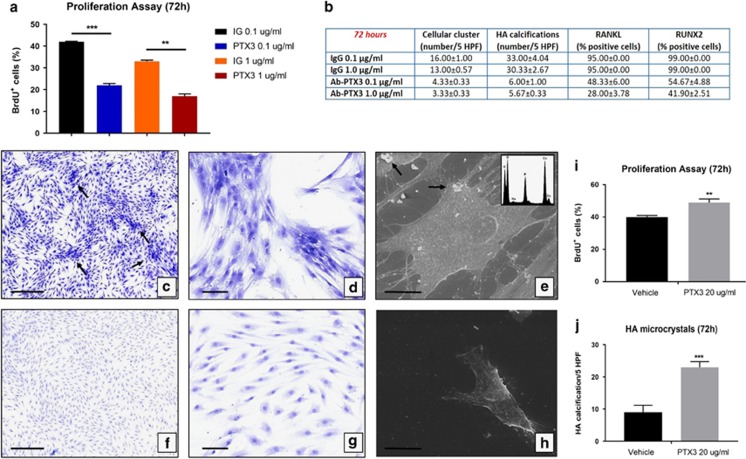
Effects of anti-PTX3 antibody on human osteoblast primary cultures. (**a**) Graph displays the percentage of proliferating osteoblasts in treated and control primary cultures (Ig 0.1 *μ*g/ml *versus* PTX3 0.1 *μ*g/ml *P*<0.0001; Ig 1 *μ*g/ml *versus* PTX3 1 *μ*g/ml *P*<0.001). (**b**) Table showed the main characteristics of primary osteoblast cultures after 72 h of treatment with PTX3 antibody (cellular cluster IgG 0.1 *μ*g/ml *versus* PTX3 0.1 *μ*g/ml *P=0.0048; IgG 1 *μ*g/ml *versus* PTX3 1 *μ*g/ml *P*=0.0501; HA microcrystals IgG 0.1 *μ*g/ml *versus* PTX3 0.1 *μ*g/ml **P=0.0017; IgG 1 *μ*g/ml *versus* PTX3 1 *μ*g/ml **P=0.0035; RANKL IgG 0.1 *μ*g/ml *versus* PTX3 0.1 *μ*g/ml *P=0.0354; IgG 1 *μ*g/ml *versus* PTX3 1 *μ*g/ml *P=0.0317; RUNX2 IgG 0.1 *μ*g/ml *versus* PTX3 0.1 *μ*g/ml *P=0.0330; IgG 1 *μ*g/ml *versus* PTX3 1 *μ*g/ml *P=0.0418). Well-differentiated osteoblasts forming numerous cellular cluster (arrows) in control cultures (1 *μ*g/ml rabbit IgG; 72 h). Scale bar represents 200 *μ*m for panel (**c**) image and 100 *μ*m for panel (**d**) image. (**e**) SEM-EDX analysis showed the presence of well-differentiated osteoblast and micro-HA calcifications (arrows) in cell cultures treated with 1 *μ*g/ml rabbit IgG (72 h). Scale bar represents 30 *μ*m. (**f** and **g**) After 72 h, the treatment with 1 *μ*g/ml of anti-PTX3 antibody induced a strong reduction of osteoblasts size and the appearance of cells with fibroblast–mesenchymal characteristics. Scale bar represents 200 *μ*m for panel (**f**) image and 100 *μ*m for panel (**g**) image. (**h**) Electron micrograph shows ultrastructural characteristics of fibroblast–mesenchymal-like cells. Scale bar represents 30 *μ*m. (**i**) rhPTX3 induce a significant increase of cell proliferation in primary osteoblast cultures derived from OP patients (*P*=0.0023) (**j**) rhPTX3 induce a significant increase of HA crystal formation in primary osteoblast cultures derived from OP patients (*P*<0.001)

To establish whether PTX3 affects osteoblast behaviour, osteoblasts derived from CTRL patients cultured for 4 weeks were seeded at a density of 30 × 10^3^ cells/well. At the beginning of culture (T0), 0.1 or 1 *μ*g/ml PTX3 antibody or rabbit IgG at the same concentrations were added to the medium, and cells were cultured for additional 72 h. Cell proliferation, morphology, micro-HA crystals and osteoblast characteristics were then evaluated. The results showed that treatment with anti-PTX3 antibody significantly inhibited cellular proliferation in a dose-dependent manner (Figure 5a), without affecting cell viability, as assessed by nuclear feature (Figure 5g) and trypan blue exclusion (data not shown). We assessed these cells for morphological and metabolic changes indicative of osteoblast de-differentiation (Figures 5c, d, f and g). Cells cultured in control conditions showed large and flattened shape, peculiar of osteoblast phenotype, and positive signal for RUNX2 and RANKL in >95% of the cells (Figure 5b). Notably, almost all the osteoblasts cultured in the presence of PTX3 antibody acquired a spindle shape, resembling fibroblast–mesenchymal cells (Figures 5f and g). In addition, the number of cells expressing both RANKL and RUNX2 significantly decreased in conditioned osteoblast cultures compared with controls (Figure 5b). Osteoblasts cultured in control conditions aggregated forming numerous cellular clusters (Figures 5c and d) and showed micron-sized HA crystals both in the cytoplasmic (nucleation vesicles) and extracellular compartment (Figure 5e) when analysed by SEM and EDX. Conversely, osteoblasts treated with anti-PTX3 antibody lost the ability to aggregate in clusters (Figures 5f and g) and showed rare or absent micro-HA crystal deposition (Figure 5h). In addition, to further elucidate the effects of PTX3 on osteoblasts behaviour, we treated osteoblast primary cultures derived from OP patients with recombinant human PTX3 (20 ng/ml) for 72 h. As shown in Figure 5, the effect of exogenous PTX3 induce a significant increase of both cell proliferation (18.37%) (Figure 5i) and formation of HA microcrystals (Figure 5j) with respect to cultures treated with vehicle. Noteworthy, already after 72 h it is possible to observe the formation of calcified nodules in osteoblast primary cultures derived from OP patients treated with recombinant human PTX3.

## Discussion

PTX3 is induced by a variety of cytokines in immune cells and has a non-redundant role in the regulation of inflammation.^22^ It acts as an extrinsic onco-suppressor gene in mouse and human by regulating complement-dependent, macrophage-sustained, tumor-promoting inflammation.^23^ Moreover, PTX3 is also produced by several cell types under appropriate stimuli and, likely owing to its complex quaternary structure, it is able to interact with several molecules, including growth factors, extracellular matrix components and fibrinolytic enzymes, thereby regulating cell proliferation and matrix remodelling in a variety of tissues.^22^ It has been recently shown that human mesenchymal cells derived by bone marrow express PTX3 when induced to differentiate *in vitro* into osteogenic lineage.^20^ It has been proposed that PTX3 elevation during bone inflammatory conditions promotes RANKL production and favour osteoclastogenic potential by osteoblasts, implying its involvement in bone resorption.^20^ We now explored the role of PTX3 in bone homeostasis by studying PTX3 expression in osteoblasts from healthy and OP patients who experience bone mass loss without apparent involvement of inflammatory stimuli. The results show a positive correlation among PTX3 expression, bone density and osteoblast proliferation and maturation, indicating that PTX3 acts as a promoter of bone deposition.

The first aim of this study was to determine whether the expression of PTX3 in human osteoblasts from OP patients differed from those from patients affected by OA, that is, of comparable age (74–76 years) but experiencing bone alterations not linked to bone density loss (surrogate control), and with those from young (from 18 to 46 years) subjects not affected by bone diseases (CTRL).

As expected, the mean value of bone morphometric parameters showed that the bone from OP patients had a significant reduction of bone mass (BV/TV and Tb.Th). Clinical analysis excluded the occurrence of osteoporosis in OA and CTRL patients enrolled. Then immunohistochemical analysis performed on bone head biopsies of young healthy patients displayed that, besides hematopoietic bone marrow cells,^22^ a high percentage of osteoblasts in the endostium expresses PTX3. This *in vivo* observation confirms and extends previous studies showing that PTX3 expressed by osteoblasts differentiated *in vitro* from bone marrow mesenchymal cells.^20^ Interestingly, the comparison of immunohistochemical results obtained by OP, OA and CTRL showed a close association between bone health and PTX3. The number of PTX3-expressing osteoblasts was positively correlated with BV/TV and Tb.Th values reaching the maximum in the CTRL group. Noteworthy, the proportion of PTX3-positive osteoblasts resulted to be significantly lower in OP patients compared with both young patients and old OA patients. In addition, the few OP patients with relative elevated number of PTX3-expressing osteoblasts also showed higher values of BV/TV and Tb.Th. Also, preliminary data indicate a positive putative association between PTX3 serum levels and bone quality in CD1 mice (data not shown); a significant reduction of BV/TV and Tb.Th were observed in concomitance with a 15% decrease of PTX3 serum levels. These results suggested an active involvement of PTX3 in bone formation.

*Ex vivo* culture of osteoblasts isolated from the three groups of patients confirmed *in vivo* observation. We found that 95% of the cells were immunopositive for the osteoblast lineage master gene RUNX2 regardless of their patient origin, but OP primary osteoblast cultures showed deep difference with the other two groups in PTX3 co-expression. Of all the RUNX2-positive cells, >75% was also positive for PTX3 in CTRL and OA primary osteoblast cultures while the number dropped to 5–10% in OP osteoblast cultures. Accordingly, western blotting analysis showed 80% reduction of PTX3 in the cell culture extracts of OP compared with CTRL and OA samples. The evidence that the deficiency of PTX3 production is maintained by OP-derived osteoblasts when cultured *in vitro* suggests that PTX3 gene is not negatively affected by environmental factors but rather that an appropriate positive signal is missing or that the PTX3 promoter is stably silenced by epigenetic mechanisms, as recently demonstrated in human cancers.^24^ That PTX3 is positively involved in bone metabolism is further supported by the evidence that cell cultures derived from OP patients displayed a poor propensity to produce mineralized matrix as demonstrated by rare presence of micro-HA crystals. Moreover, the treatment of human osteoblast primary cultures derived from CTRL patients with anti-PTX3 antibody induced considerable changes in osteoblast behaviour. Specifically, they lost the morphological and molecular features typical of mature osteoblasts, acquiring fibroblast-like shape and drastically decreasing RANKL and RUNX2 expression. Notably, the inhibition of PTX3 negatively affected osteoblast’s proliferation and their ability to form both cellular clusters and micro-HA crystals. In addition, we reported the effects of exogenous PTX3 on osteoblast primary cultures derived from OP patients. Our data clearly demonstrated the ability to PTX3 to induce an increase of both cell proliferation and HA microcrystal formation in osteoblasts characterized by no/low expression of PTX3. These results suggest a central role of PTX3 in osteoblast proliferation, differentiation and function. In this context, FGF2 could be a key mediator of the relationship between bone metabolism and PTX3. It is known that PTX3 contains two FGF2-binding sites and sequesters this growth factor thereby inhibiting its action on target cells.^25^ This interaction can modulate the capability of FGF2 to interfere with osteoblast activity. Indeed, although FGF2 is generally considered to favour bone deposition, it exerts differentiation-stage-specific effects on osteoblasts.^26^ Thus, the ability of PTX3 to sequester FGF2 via its N-terminal extension can influence the osteoblastogenesis by regulating the activity of FGF2.

The correlation between osteoblast PTX3 deficiency and low bone density reported here in humans well matches with bone structure deficiency and impaired fracture healing observed in the animal model of PTX3 null mice.^21^

The requirement of fine control of PTX3 expression for maintaining the bone in good health is also strengthened by the studies performed in conditions mimicking *in vivo* and *in vitro* acute inflammation.^20^ In such inflamed situations, cytokines induced PTX3 overexpression by osteoblasts, which in turn increased their osteoclastogenic potential by elevating RANKL production. Altogether, these results allow to hypothesize an opposite effect of PTX3 in the delicate balance of bone remodelling depending on health conditions. In physiological conditions, the expression of PTX3 would stimulate osteoblast differentiation by bone marrow mesenchymal stem cells. On the other hand, a decreased PTX3 production, as that observed in OP patients, could result in inadequate bone formation and an excessive PTX3 elevation in inflammatory conditions, such as in rheumatoid arthritis, could promote bone resorption, in both cases leading to bone mass loss.

### Limits of the study

The evaluation of PTX3 was performed using a semiquantitative approach (immunohistochemistry), as protein or mRNA extraction is difficult to perform on bone biopsies. In addition, it is not always possible to obtain suitable material from clinical sources. To corroborate our results, western blotting analysis was performed on primary osteoblast cultures derived from OP, OA and CTRL patients. In our laboratory, blood serum concentration of creatinine, nitrogen (BUN), phosphorus, calcium, Vit D (25OHD3) and intact parathyroid hormone (PTH) were not assessed in patients undergoing hip arthroplasty for high-energy hip fractures. Nevertheless, the bone quality of all these patients was evaluated by histomorphometric analysis (Figure 2). Unfortunately, it is very difficult to collect bone head biopsies of patients aged >70 years without OP or OA who underwent hip arthroplasty for high-energy hip fractures. However, the results demonstrated that OA patients were good surrogate controls in this study, showing several similarities with younger patients.

## Conclusions

The identification of new determinants of bone loss in osteoporosis is a field in constant development. This study suggests an important role of PTX3 in normal bone homeostasis showing its involvement in osteoblast proliferation, differentiation and function. Its impaired expression by osteoblast cells in OP patients strongly support the hypothesis, that PTX3 is a novel regulator of bone metabolism with prominent effects on cellular processes that are essential for normal bone physiology. Further studies are needed to elucidate the molecular mechanisms through which PTX3 regulates the activities of osteoblasts.

## Materials and methods

All experiments described in the present study were approved by the ethics committee of ‘Policlinico Tor Vergata’ (approval reference number 85/12). All experimental procedures were carried out according to The Code of Ethics of the World Medical Association (Declaration of Helsinki). Informed consent was obtained from all patients prior to surgery. Specimens were handled and carried out in accordance with the approved guidelines.

### Patients

We enrolled 65 patients who underwent hip surgery in the Orthopaedic Department of ‘Tor Vergata’ University Hospital in the period June 2014–February 2015. We enrolled 25 consecutive patients who underwent hip arthroplasty for medial hip fractures for low-energy trauma (18 women and 7 men; 76.65±1.44 years), and 25 consecutive patients who underwent hip arthroplasty for OA (15 women and 10 men; 74.21±1.26 years). Moreover, we enrolled 15 patients (7 women and 8 men; 46.19±2.78 years) who underwent hip arthroplasty for high-energy hip fractures (control group) (Table 1).

Exclusion criteria were history of cancer, myopathies or other neuromuscular diseases or chronic administration of corticosteroid for autoimmune diseases (>1 month), diabetes, alcohol abuse and HBV, HCV or HIV infections.

### Bone mineral density evaluation (DXA)

DXA was performed with a Lunar DXA apparatus (GE Healthcare, Madison, WI, USA). Lumbar spine (L1–L4) and femoral (neck and total) scans were performed, and bone mineral density (BMD) was measured according to the manufacturer’s recommendations.^27^ Dual-energy X-ray absorptiometry measures BMD (in grams per square centimetre), with a coefficient of variation of 0.7%. For patients with fragility fractures, BMD was measured on the uninjured limb. For all the other patients, measurements were performed on the non-dominant side, with the participants supine on an examination table with their limbs slightly abducted.^28^ DXA exam was performed 1 day before surgery for OA patients and 1 month after surgery for OP and control patients (CTRL). The results were expressed as *T*-scores.

### Radiographic analysis

Anteroposterior radiographs of the pelvis of all groups were obtained using a standard, validated protocol.^29^ Two orthopaedists independently assessed all radiographs using the K–L radiographic atlas.^30^ Patients with a grade of K–L≥2 were considered osteoarthritic.

### Blood tests

Blood serum concentration of creatinine, nitrogen (BUN), phosphorus, calcium, Vit D (25OHD3) and intact PTH were assessed.

### Sampling

At surgery, the femoral head was removed to implant prosthesis. Bone samples were taken for histological analysis, excluding areas with macroscopic alterations of trabecular bone such as necrotic areas.

### Histology

Bone biopsies of the femoral head were fixed in 4% paraformaldehyde for 24 h and paraffin embedded without decalcification.^31^ From each patient, we obtained two paraffin blocks. Undecalcified tissues were cut by a tungsten carbide knife; 3 *μ*m-thick sections were stained using H&E.

### Histomorphometric analysis

Ten microscopic images, randomly selected, were evaluated for each biopsy sample. Images were acquired at × 40 magnification using a Nikon Eclipse E600 light microscope connected to a Nikon digital camera (Nikon Corp, Japan) and saved at a resolution of 1280 × 1024 pixels. Image analysis was performed using a BioQuant Osteo software (version7.20.10; BIOQUANT Image Analysis Corporation, Nashville, TN, USA) according to the manufacturer’s instructions.^32^ The following parameters: BV/TV, Tb.Th, and Tb.S, were evaluated according to Dempster *et al.*^33^

Moreover, H&E slides were analysed using the Viewing software (Ventana, Tucson, AZ, USA) to evaluate bone tissue composition (trabecular, bone marrow and fat).

### Immunohistochemistry

RUNX2, VdR, OPG, sclerostin and PTX3 expression were assessed in femoral head biopsies by immunohistochemistry. Briefly, 3 *μ*m-thick sections were pretreated with EDTA citrate pH 7.8 for 30 min at 95 °C and then incubated with mouse monoclonal anti-RUNX2 antibody for 60 min (1 *μ*g/ml, clone EPR14334, AbCam, Cambridge, UK), rabbit monoclonal Anti-VDR for 60min (1 *μ*g/ml, clone SP141, Spring Bioscience, CA, USA), rabbit polyclonal anti-sclerostin antibody for 60 min (1 *μ*g/ml, clone NA, AbCam), mouse monoclonal Anti-OPG for 60 min (1 *μ*g/ml, clone 98A1071, Novus Biologicals, Littleton, CO, USA). For PTX3 evaluation, sections were pretreated with citrate pH 6 for 30 min at 95 °C and then incubated with rat monoclonal anti-PTX3 for 120 min (2 *μ*g/ml, clone MNB1, AbCam). Washings were performed with PBS/Tween20 pH 7.6 (UCS Diagnostic, Rome, Italy); reactions were revealed by horseradish peroxidase (HRP)-3,3' diaminobenzidine (DAB) Detection Kit (UCS Diagnostic). To assess the background of immunostaining, we included a negative control for each reaction by incubating the sections with secondary antibodies (HRP) and a detection system (DAB).

#### Human osteoblast primary cell cultures

Primary cultures of osteoblasts were obtained from the cancellous bone of: patients with high-energy femoral fracture (CTRL, Caucasian, 18 years), patients affected by osteoporosis (OP, Caucasian, 71 years), and patients affected by osteoarthrosis (AO, Caucasian, 72 years). The samples were dissected and treated to obtain a homogeneous population of osteoblasts. Briefly, after dissection, trabecular bone fragments were repeatedly washed in PBS. Then bone fragments were briefly incubated at 37 °C with 1 mg/ml Trypsin from porcine pancreas ≥60 /mg (SERVA Electrophoresis GmbH, Heidelberg, Germany) diluted in DPBS. After washing, bone fragments were subjected to repeated digestions with 2.5 mg/ml Collagenase NB 4G Proved grade ≥0.18 U/mg (SERVA Electrophoresis GmbH) diluted in DPBS with calcium and magnesium. Supernatant were collected and centrifuged at 310 RCF for 5 min. Cell pellets were resuspended in DMEM with 15% FBS, seeded into a 24-well plate and incubated at 37 °C, 5% CO_2_ until reaching confluence (about 4 weeks). Medium was changed twice a week. Osteoblasts were characterized by alkaline phosphatase test and immunostained for RUNX2 and RANKL.

#### Immunostaining of primary cell cultures

Expression of RUNX2 and PTX3 was simultaneously evaluated by dual-colour immunofluorescence in confluent CTRL, OP and OA primary osteoblast cultures. Briefly, after fixation in PFA 4% for 30 min, cell cultures were pretreated with EDTA citrate pH 7.8 for 5 min at 95 °C and incubated with mouse monoclonal anti-RUNX2 antibody for 30 min (1 *μ*g/ml, clone EPR14334, AbCam). Reaction with anti-Runx2 was revealed by using FITC-conjugated anti-mouse antibody. Afterwards, cell cultures were incubated with rat monoclonal anti-PTX3 (2 *μ*g/ml, clone MNB1, AbCam) for 30 min. Reaction with anti-PTX3 was revealed by using Texas Red-conjugated anti-rat antibody. Washing was performed with PBS/Tween 20 pH 7.6 (UCS Diagnostic).

### Western blotting analysis

To detect PTX3 in osteoblast primary cultures derived by OP (*n*=3), OA (*n*=3) and CTRL (*n*=3) patients, we performed a western blotting analysis. Cell proteins extracted by using RIPA buffer were separated by 4–15% precast SDS-PAGE (Bio-Rad, Hercules, CA, USA) under reduced conditions. Protein concentration was determined using the Pierce BCA Protein Assay Kit (Thermo Scientific, Vilnius, Litheania). Equal amounts of protein (20 *μ*g) were resolved on 8–16 % SDS-PAGE and transferred to nitrocellulose membrane. Then membranes were incubated with a rat monoclonal antibody against human PTX3 (1 *μ*g/ml, clone MNB1, AbCam) and successively with anti-rat IgG coupled to HRP. Inmunoreactive electrophoretic bands were detected by enhanced chemiluminiscence ECL Advance, Amersham (GE Healthcare Life Sciences, Little Chalfont, Buckinghamshire, UK) using a VersaDoc 5000 Imager (Bio-Rad) and quantified by the Quantity One software (Bio-Rad). Relative amounts of PTX3 were normalized for the corresponding *β*-actin values from cell lysates.

### Real-time PCR

RNA was isolated from cells by using Qiazol reagent (Qiagen,Hilden, Germany), and 1 mg of RNA was reverse-transcribed into cDNA by using the RevertiAid First Strand cDNA Synthesis Kit (Thermo Scientific). Quantitative real-time PCR was performed by using a Power SYBR Green 1-Step Kit and the ABI 7000 Real Time PCR System (Applied Biosystems, Carlsbad, CA, USA) according to the manufacturer’s instructions.^34^ The sequences of the primers used were: PTX3, 5′-TTTTGGAAGCGTGCATCCTGT-3′ (sense) and 5′-CACCACCAACACTAGGGACTG-3′ (antisense).

### Osteoblast primary cultures conditioned with anti-PTX3 antibody

To evaluate the role of PTX3 in osteoblast activity, we treated primary osteoblast cultures from CTRL patient with anti-PTX3 antibody (clone MNB1, AbCam). In detail, cells from the first or second passage were seeded into a 24-well plate at a density of 30 × 10^3^ cells/well. Successively, human osteoblast cells were treated with: (a) Rabbit anti-mouse IgG 0,1 *μ*g/ml (CTRL), (b) Rabbit anti-mouse IgG 1 *μ*g/ml (CTRL), (c) mouse monoclonal anti-PTX3 (clone MNB1) 0,1 *μ*g/ml, and (d) mouse monoclonal anti-PTX3 (clone MNB1) 1 *μ*g/ml. Cell proliferation, morphology, micro-HA crystals and osteoblast characteristics were evaluated at time 0, 6, 24, 48 and 72 h. Cell proliferation was investigated by both counting the number of cells for each time point and bromodeoxyuridine Incorporation Assay performed at time 0 and after 72 h. Morphology was studied by both toluidine blue staining and SEM analysis. Identification of micro-HA crystals was performed by SEM-Energy Dispersive X-ray (EDX) microanalysis, whereas osteoblast markers were studied by immunofluorescence for RUNX2 (1 *μ*g/ml, clone EPR14334, AbCam) and RANKL (mouse monoclonal anti-RANKL, 1 *μ*g/ml, clone 12A380).

### Osteoblast primary cultures of OP conditioned with recombinant human PTX3

To further elucidate the functions of PTX3 on osteoblast behaviour, we examined the effects of exogenous PTX3 on osteoblasts derived from OP patients by culturing it with 20 ng/ml of recombinant human PTX3 for 72 h. In detail, cells from the first or second passage were seeded into a 24-well plate at a density of 30 × 10^3^ cells/well. Successively, human osteoblast cells were treated with: (a) 20 ng/ml recombinant human PTX3 and (b) Vehicle (equal amount of PBS).

Cell proliferation was investigated by bromodeoxyuridine Incorporation Assay performed at time 0 and after 72 h. Identification of micro-HA crystals was performed by SEM-EDX microanalysis.

### Statistical analysis

All statistical analyses were performed using the GraphPad Prism 5 Software (GraphPad Prism, La Jolla, CA, USA).

Clinical data were analysed by Mann–Whitney test; immunohistochemical and morphometric parameters were analysed by one-way ANOVA and Mann–Whitney test. Cell growth, cellular clusters, micro-HA calcifications and both RUNX2 and RANKL expression of primary osteoblasts cultures was analysed by one-way ANOVA and *t*-test.

## Figures and Tables

**Table 1:** Main characteristics of OP, OA and CTRL patients

	**OP**	**OA**	**CTRL**	***T*****-test (Mann–Whitney test)**
Age, years	76.65±1.44	74.21±1.26	46.19±2.78	OP *versus* OA NS (*P*=0.11); OP *versus* CTRL *** (*P*<0.001); OA *versus* CTRL *** (*P*<0.001)
BMI	22.35±1.37	27.06±0.50	—	*** (*P*=0.0005)
T Score (L1–L4)	−2.9±0.13	0.66±0.3	0.85±0.01	*** (*P*=0.0005)
T Score (neck)	−2.8±0.18	−0.1±0.12	0.15±0.08	*** (*P*=0.0005)
Creatinine (BUN) (mg/dl)	0.88±0.12	1.70±0.24	—	NS (*P*=0.15)
Azotemia (mg/dl)	50.01±3.5	48.20±4.10	—	NS (*P*=0.70)
Phosphorus (mg/dl)	2.75±0.10	3.20±0.28	—	** (*P*=0.0073)
Calcium (mg/dl)	7.99±0.18	9.33±0.14	—	NS (*P*=0.34)
Vit D(25OHD3) (ng/ml)	10.22±2.61	23.63±8.00	—	NS (*P*=0.48)
PTH (pg/ml)	123.10±44.08	92.00±12.08	—	NS (*P*=0.56)

**Table 2:** Scoring system for immunohistochemical analysis

**Score**	**0**	**1**	**2**	**3**
Positive cells	≤2	3≤*x*≤12	13≤*x*≤22	≥23
